# The effect of a pharmaceutical transitional care program on rehospitalisations in internal medicine patients: an interrupted-time-series study

**DOI:** 10.1186/s12913-019-4617-9

**Published:** 2019-10-21

**Authors:** Fatma Karapinar-Çarkıt, Sander D. Borgsteede, Marjo J. A. Janssen, Marlies Mak, Nimet Yildirim, Carl E. H. Siegert, Peter G. M. Mol, Toine C. G. Egberts, Patricia M. L. A. van den Bemt

**Affiliations:** 1 Department of Clinical Pharmacy, OLVG, Jan Tooropstraat 164, 1061AE Amsterdam, the Netherlands; 2 Health Base, Department of medication surveillance, Papiermolen 36, 3994 DK Houten, The Netherlands; 3 Department of Internal Medicine, OLVG, Amsterdam, The Netherlands; 4 Department of Clinical Pharmacology, University Medical Centre Groningen, Groningen, The Netherlands; 5 Department of Clinical Pharmacy, University Medical Centre Utrecht, Utrecht, The Netherlands; 6 Utrecht Institute for Pharmaceutical Sciences, Division of Pharmacoepidemiology & Clinical Pharmacology, Faculty of Science, Utrecht University, Utrecht, The Netherlands; 7 Department of Clinical Pharmacy and Pharmacology, University Medical Center Groningen, PO Box 30.001, 9700 RB Groningen, The Netherlands

**Keywords:** Patient discharge, Continuity of care, Medication reconciliation, Patient education, Medication errors, Hospital readmission

## Abstract

**Background:**

Medication errors at transition of care can adversely affect patient safety. The objective of this study is to determine the effect of a transitional pharmaceutical care program on unplanned rehospitalisations.

**Methods:**

An interrupted-time-series study was performed, including patients from the Internal Medicine department using at least one prescription drug. The program consisted of medication reconciliation, patient counselling at discharge, and communication to healthcare providers in primary care. The primary outcome was the proportion of patients with an unplanned rehospitalisation within six months post-discharge. Secondary outcomes were drug-related hospital visits, drug-related problems (DRPs), adherence, believes about medication, and patient satisfaction. Interrupted time series analysis was used for the primary outcome and descriptive statistics were performed for the secondary outcomes.

**Results:**

In total 706 patients were included. At 6 months, the change in trend for unplanned rehospitalisations between usual care and the program group was non-significant (− 0.2, 95% CI -4.9;4.6). There was no significant difference for drug-related visits although visits due to medication reconciliation problems occurred less often (4 usual care versus 1 intervention). Interventions to prevent DRPs were present for all patients in the intervention group (mean: 10 interventions/patient). No effect was seen on adherence and beliefs about medication. Patients were significantly more satisfied with discharge counselling (68.9% usual care vs 87.1% program).

**Conclusions:**

The transitional pharmaceutical care program showed no effect on unplanned rehospitalisations. This lack of effect is probably because the reason for rehospitalisations are multifactorial while the transitional care program focused on medication. There were less hospital visits due to medication reconciliation problems, but further large scale studies are needed due to the small number of drug-related visits. (Dutch trial register: NTR1519).

## Related publications directly to this study

- Study protocol: Karapinar-Carkit F, Borgsteede SD, Zoer J, Siegert C, Van TM, Egberts AC, et al. The effect of the COACH program (Continuity Of Appropriate pharmacotherapy, patient Counselling and information transfer in Healthcare) on readmission rates in a multicultural population of internal medicine patients. BMC Health Serv Res. 2010;10:39
- Karapinar-Carkit F, et al. Cost-effectiveness of a transitional pharmaceutical care program for patients discharged from the hospital. PLoS One. 2017;12(4):e0174513.

## Background

Medication errors occur frequently at transition of care and can negatively affect patient safety [1]. Four key factors contribute to these errors. The first factor is the lack of complete sources to assess patients’ medication use. In combination with patients’ recall bias this results in incorrect prescriptions at hospital admission [2]. These admission errors can carry over to the discharge medication. The second factor is insufficient evaluation of the pharmacotherapy. For example, when temporarily discontinued medication is forgotten and not restarted (e.g. anticoagulants). Or medication intended for temporary use are continued (e.g. hypnotics, proton-pump inhibitors) [3]. The third factor is insufficient patient involvement. Hospitalised patients often get help with the administering of their medication by hospital staff. After hospital discharge, patients are abruptly expected to manage their medication themselves, generally with little preparation [4]. The last factor regards insufficient communication from hospital to primary care. Discharge letters and prescriptions generally do not contain the entire pharmacotherapy and changes therein [5, 6]. Both the general practitioner and community pharmacy lack information of reasons for all changes, making it unclear whether changes should be maintained, were only temporary or were unknown to hospital staff [7, 8].

Transitional care programs, focusing on the transition from hospital to the community setting, have been developed. Evidence exists that interventions around discharge medication can reduce adverse events, reduce rehospitalisations and improve adherence [9–14]. However, some studies showed no effect and Holland et al. reported contradictory results on the rate of rehospitalisations [15–17]. Most studies have implemented single interventions using educational strategies or medication reconciliation [9, 11, 18–23]. However, to address all four key factors, as stated above, requires multiple interventions to affect transition of care-related medication errors.

Therefore, the COACH (Continuity Of Appropriate pharmacotherapy, patient Counselling and information transfer in Healthcare) program has been designed by combining interventions. The aim of this study is to determine the effect of the COACH program on unplanned rehospitalisations within six months after discharge from an Internal Medicine department.

## Methods

### Design

This was a prospective interrupted time-series study at a general teaching hospital; OLVG (formerly Sint Lucas Andreas Hospital), Amsterdam, The Netherlands. This is a quasi-experimental study that is characterized by a series of measurements over time interrupted by an intervention [24]. We regarded a randomized design as not feasible, because we changed how care was organised and previous experiences with pilot projects have shown that the COACH program contaminates usual care as residents and other healthcare providers learn from the COACH program. The program therefore influences prescribing behaviour. Therefore, we have chosen for a study with a before-after design including interrupted time series as the preferred alternative.

**Fig. 1:**
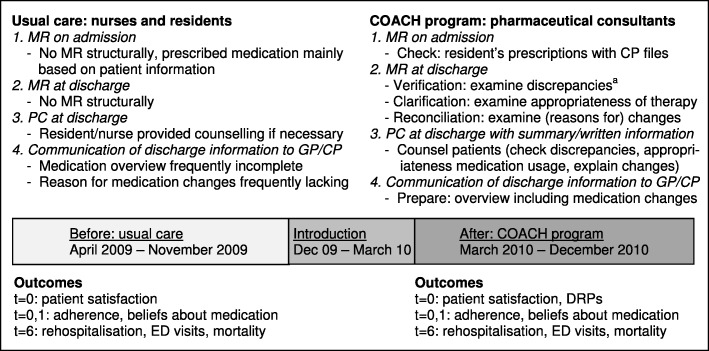
Timeline of the COACH program and of the introduction and implementation of the program. ^a^ discrepancies between medication prescribed pre-admission and medication prescribed in the hospital. CP = community pharmacy, DRPs = drug-related problems, ED = emergency department, GP = general practitioner, PC = patient counselling, MR = medication reconciliation, t = 0,1,6: respectively, at discharge,1 month after discharge and 6 months after discharge

During eight months usual care patients were included (Fig. 1). During an intermediate period of 3.5 months the COACH program was introduced. Patients were again included during a nine month post-intervention period, once the program had settled in. The study protocol has been described elsewhere [25].

This study was submitted to the Institutional Medical Ethics Committee of the Sint Lucas Andreas Hospital and exempted from review by the committee as this is not required for studies that do not affect the patient’s integrity (according to Dutch legislation). Patient data were obtained and handled in accordance with privacy regulations. Patients provided written informed consent for the study (reference number of the study mec09/005).

### Study population

All admitted patients to the Internal Medicine department with at least one prescribed drug intended for chronic use were invited to participate. Exclusion criteria were: no informed consent, no medication prescribed at discharge, died during index admission, lived outside the catchment area of the hospital (as we were unable to obtain rehospitalisation data), transfer to another department, hospital or nursing home (because these patients are not responsible for medication use themselves), discharge within 24 h or out of office hours, impossibility to counsel (as stated by the resident due to physical/mental constraints, being critically ill or due to language restrictions without relatives or healthcare personnel to translate). Patients could be included in the study only once.

### Usual care

At hospital admission and discharge, medication reconciliation was not performed structurally (Fig. 1). Residents mostly used the information provided by patients, carers, or previous hospital records to prescribe the medication. Residents could consult community pharmacy medication records. A clinical pharmacist checked prescribed medication for correct dosages, the presence of double medication, or potential drug-drug interactions and contra-indications using the Computerized Physician Order Entry (CPOE) system.

Residents and nurses performed patient counselling at hospital discharge to explain medication changes if regarded necessary. Discharge medication information was communicated to the general practitioner (GP) and community pharmacy. This communication contained little or no information on (reasons for) changes in the pharmacotherapy.

In the Netherlands, community pharmacists and general practitioners are frequently linked to each other. When a community pharmacist changes a medication record in their information system, this information is automatically communicated electronically to the general practitioner.

### COACH intervention program

A team of pharmaceutical consultants carried out the COACH program with clinical pharmacists as supervisors. Pharmaceutical consultants are specialized pharmacy technicians who have followed an additional three year bachelor program focusing on pharmaceutical patient care. Therefore, they are educated in medication errors and communication with patients. In the hospital they have followed a training program to perform medication reconciliation.

At hospital admission and discharge, medication reconciliation was performed by verifying the admission and discharge prescriptions of the resident in the hospital’s CPOE with community pharmacy records and assessing patient information. Discrepancies with the pre-admission medication and possible drug-related problems were communicated to the resident using a protocol [25]. The resident adjusted the prescriptions if necessary.

At hospital discharge, the pharmaceutical consultant counselled the patient/carer using a medication summary that contained all known pharmacotherapy and (reasons for) medication changes. The same information was faxed to the community pharmacy before discharge. The resident could upload this information into the discharge letter for the general practitioner. Every (new) resident was trained in the research protocol and the study flow was presented on a poster in their office.

### Study endpoints and data collection

The primary outcome was the proportion of patients with at least one unplanned rehospitalisation within six months after discharge. An unplanned rehospitalisation was defined as an unscheduled hospitalisation, which occurred after discharge, to the OLVG Hospital or any other hospital within the catchment area. Other hospital contacts, i.e. planned rehospitalisations and emergency department visits, and mortality were regarded as secondary outcomes. These data were manually collected using the hospital information systems of OLVG and five other hospitals.

Exploratory outcomes included the interventions performed to prevent drug-related problems (DRPs), adherence to drug treatment, patients’ attitude towards drugs, patients’ satisfaction with information about medicines and patients’ general satisfaction with counselling. Interventions performed to prevent DRPs were extracted from the checklists used by pharmaceutical consultants and classified according to a previously described classification system [26].

Before discharge, patients were requested to fill out validated questionnaires with a 5-point Likert scale about their adherence to drug treatment (MARS; Medication Adherence Rating Scale), their attitude towards drugs (BMQ; Beliefs about Medicines Questionnaire), satisfaction with information about medicines (SIMS) and their general satisfaction with counselling [27–31]. After one month, a second short questionnaire with MARS and BMQ was sent. Patients were phoned if they had given informed consent to fill out questionnaires but failed to respond (three attempts).

Also, a post-hoc analysis was performed to assess the proportion of patients with drug-related hospital visits. A drug-related visit was defined as any admission or emergency department visit related to the use of a drug. An Internist and a Hospital pharmacist/Clinical pharmacologist assessed whether all revisits (*n* = 424) were drug-related and whether these readmissions were potentially preventable using a blinded consensus method [32].

From the hospital information system we extracted baseline characteristics including gender, age, co-morbidities, length of stay, and previous hospital contacts in the six months before inclusion. The Charlson co-morbidity score was used to evaluate the severity of co-morbidities [33]. This score was previously associated with hospitalisations [33, 34].

Fidelity of the intervention (i.e. whether all parts of the intervention are implemented as planned) was also assessed manually. The number of paper checklists that pharmaceutical consultants used to perform medication reconciliation at hospital admission and discharge and patient counselling at hospital discharge were counted. For the information exchange to the community pharmacist we counted the number of discharge medication overviews. For the information exchange to the general practitioner, we checked whether the residents uploaded the discharge medication information, prepared by the pharmaceutical consultant, into the discharge letter.

### Sample size

Results of previous studies into pharmacist pre-discharge medication reconciliation combined with patient counselling vary widely [11, 12, 14, 35–37]. Four studies report an absolute decrease of rehospitalisation frequency of 13–30% and two studies report 5–9% (median 15%). However, the populations in these studies are not fully comparable: previous studies were limited to elderly patients and our study also included younger patients. Therefore, a conservative approach was used: 20% of rehospitalised patients in usual care and 12% in the intervention group (8% absolute reduction). With a type 1 error of 0.05, a power of 80%, a total of 360 patients per group was needed.

### Data analysis

Patients were compared using the independent t-test for continuous variables and the chi-square test for frequencies. For the interrupted time series analyses we collected data over an 8-month period with usual care and over a 9-month intervention-period with the COACH program in place. The data points for the time-series were aggregated per four weeks. For example, for unplanned rehospitalisations the number of patients with an unplanned rehospitalisation was divided by the total number of patients included in that data point. As there was only a small number of patients included in the last month in both periods, these patients were added to the previous month. Thus, there were 7 data points for the usual care-period and 8 data points for the intervention-period. The study design met EPOC criteria for a robust interrupted time series analysis, that is at least three data-points before and after the intervention, each consisting of at least 30 patients [38]. Segmented linear regression analysis was used to assess a trend for the percentage of patients with above mentioned outcomes. Durbin-Watson statistics and visual inspection of the residuals versus time were used to check for possible autocorrelation (serial correlation between an outcome and consecutive observations, non-significant Durbin-Watson means no autocorrelation). To estimate the level and trend of the outcomes in the usual care-period and to estimate the changes in level and trend after the implementation of the COACH program, the following linear regression model was used [24].

Yt= β_0_ + β_1_ * time_t_ + β_2_ * intervention_t_ + β_3_ * time after intervention_t_ + e_t_

β_0_ = usual care level of the outcome (value at time zero)

β_1_ = slope prior to the intervention (usual care trend)

β_2_ = change in outcome immediately after the intervention

β_3_ = change in the slope from before to after the intervention

Potential confounders were added to this model to evaluate the impact of imbalances in the case-mix in the usual care- and intervention-period.

Descriptive and comparative statistics (i.e. t-test, chi-squared test) were performed for the other outcomes as described in previous studies [26–31].

## Results

**Fig. 2:**
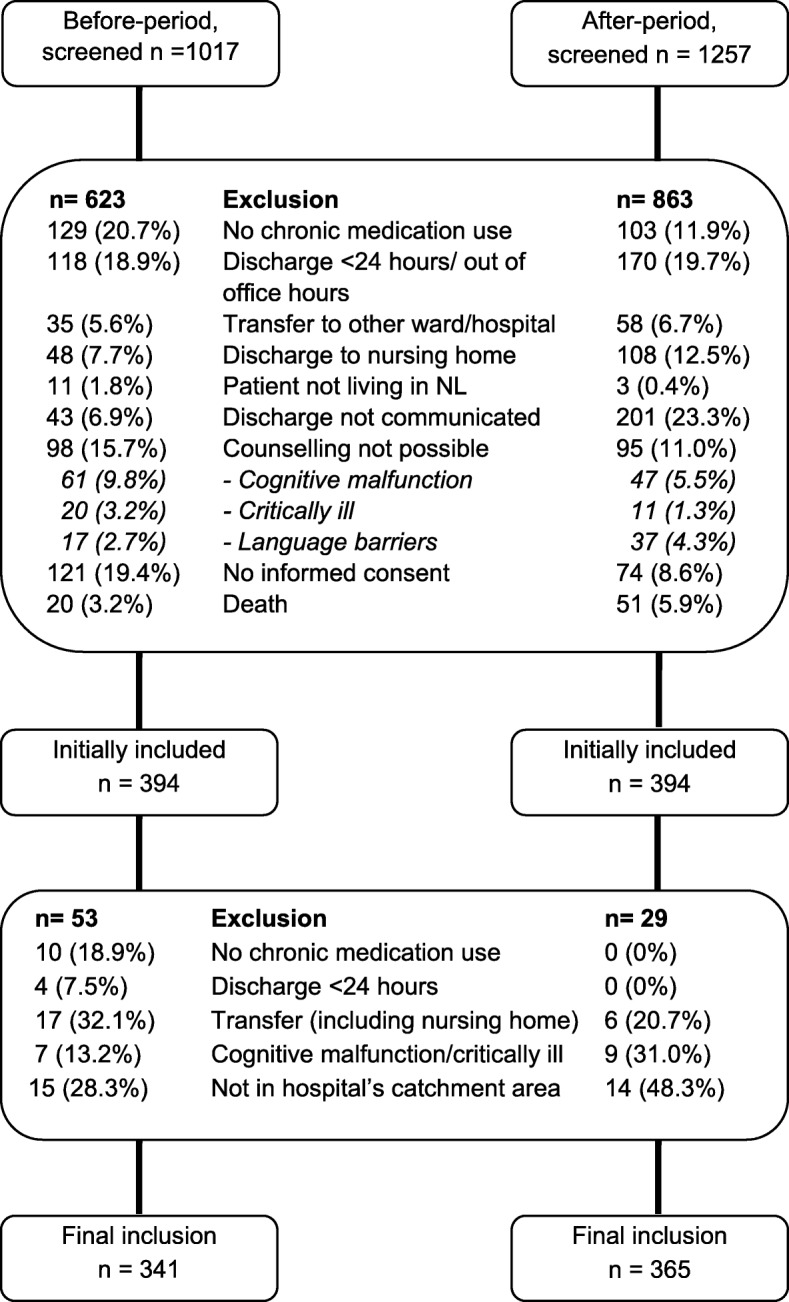
Flowchart of inclusion of patients participating in the usual care- and intervention-period

A total of 2274 patients were screened; 1568 (69%) patients were excluded (Fig. 2), leaving 706 patients (341 usual care, 365 COACH program) who were included. The main reason for exclusion were discharge within 24 h or out-of-office hours (19.4%), transfer (16.8%) and no medication use (15.6%).

Patients who did not give informed consent were significantly older (68.7 vs 65.5 years, *p* = 0.02) and stayed, non-significantly, longer in hospital (11.2 vs 9.3 days, *p* = 0.20). No difference was found for type of admission (planned/unplanned) and gender.

The patients in the usual care- and intervention-period differed in baseline characteristics (Table 1). Patients in the COACH program received more frequently help with their medication use (18.8% vs 30.8%, *p* < 0.01), had more hospital contacts before inclusion (1.3 vs 1.7, *p* = 0.03) and had a higher number of co-morbidities (3.4 vs 3.9, p < 0.01) which were also more severe (p < 0.01).

**Table 1:**
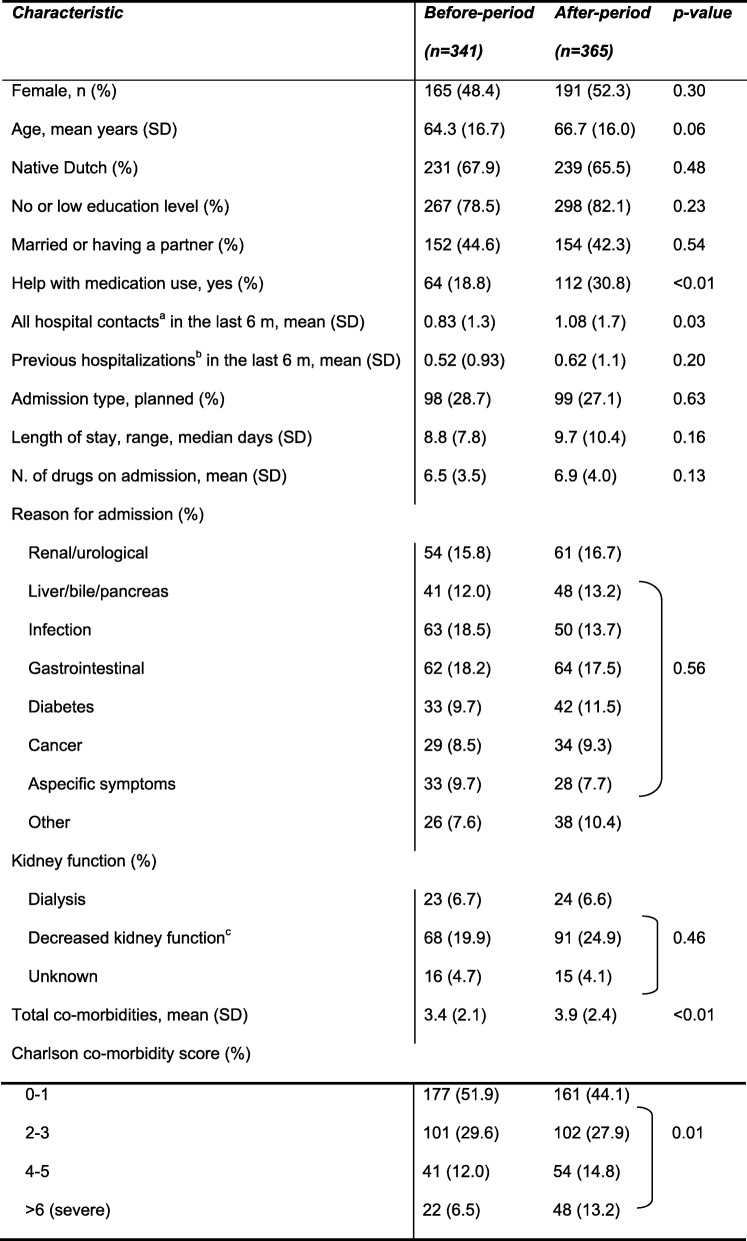
Characteristics of patients participating in the before- and after-period

^a^includes one-day care, emergency department visits, planned and unplanned admissions in the last 6 months before inclusion

^b^includes planned and unplanned admissions in the last 6 months before inclusion

^c^kidney function less than 60 ml/min during at least 3 months

### Fidelity of the COACH intervention

At hospital admission and discharge, respectively, 91.8 and 100% of patients received medication reconciliation, 100% received patient counselling at discharge and for 100% medication related information was transferred to community pharmacies (Table 2). At admission, in 8.2% of patients medication reconciliation was not performed due to a short hospital stay and medication reconciliation was therefore performed at discharge.

**Table 2:** Fidelity of the COACH program (*n* = 365)

Implementation of	After-period (%)	Performed by
Medication reconciliation at hospital admission^a^	335 (91.8)	Pharmaceutical consultant
Medication reconciliation at hospital discharge	365 (100.0)	Pharmaceutical consultant
Patient counselling at hospital discharge	365 (100.0)	Pharmaceutical consultant
Information exchange to community pharmacist	365 (100.0)	Pharmaceutical consultant
Information exchange to general practitioner^b^	102 (27.9)	Resident

^a^for the other 8.2% of patients medication reconciliation could not be performed due to a short hospitalisation

^b^for 72.1% of patient the resident failed to upload the discharge medication overview into the discharge letter. If the discharge medication overview was uploaded, the resident could adjust the information, e.g. delete information regarding allergies or contra-indications

For 102 patients (27.9%) the residents uploaded the information prepared by the pharmaceutical consultant, the reconciled discharge medication overview, into the discharge letter for general practitioners. However, 48 (13.2%) contained the exact same information as was communicated to the patient and community pharmacists. Resident for example deleted information regarding allergies or reasons for medication changes.

### Unplanned rehospitalisations

The proportion of patients with an unplanned rehospitalisation was 27.3% in the usual care vs 33.2% with the COACH program in place. The Durbin Watson statistics was not indicative for autocorrelation. In the unadjusted segmented linear regression model the baseline trend showed a non-significant decrease in unplanned rehospitalisations (i.e. β_1_, − 1.7, 95% CI -4.8; 1.4) in the usual care-period (Table 3). The introduction of the COACH program was followed by a non-significant increase of unplanned rehospitalisations (i.e. β_2_, 8.5, 95% CI -8.4; 25.5) and no change in trend (i.e. β_3_, 2.3% rehospitalisations per 4 week period 95% CI − 1.7; 6.3).

**Table 3:** Effect of COACH program on unplanned rehospitalisations (*n* = 341 before and n = 365 after)

ITS unplanned rehospitalisation	Unadjusted	Adjusted^a^
*β*_*0*_*,* usual care level of the outcome (95% CI)	34.0 (20.2; 47.9)	11.3 (− 28.7; 51.2)
*β*_*1*_*,* baseline trend (95% CI)	-1.7 (− 4.8; 1.4)	−2.1 (− 5.2; 1.1)
*β*_*2*_*,* change in outcome immediately after the intervention (95% CI)	8.5 (−8.4; 25.5)	12.7 (−7.3; 32.7)
*β*_*3*_, change in the slope from before to after the intervention(95% CI)	2.3 (−1.7; 6.3)	−0.2 (−4.9; 4.6)

*ITS* interrupted time series analysis. β values were calculated using segmented regression analysis

^a^Adjusted for baseline differences: help with medication use, all hospital contacts in the last 6 months, mean Charlson score

**Fig. 3:**
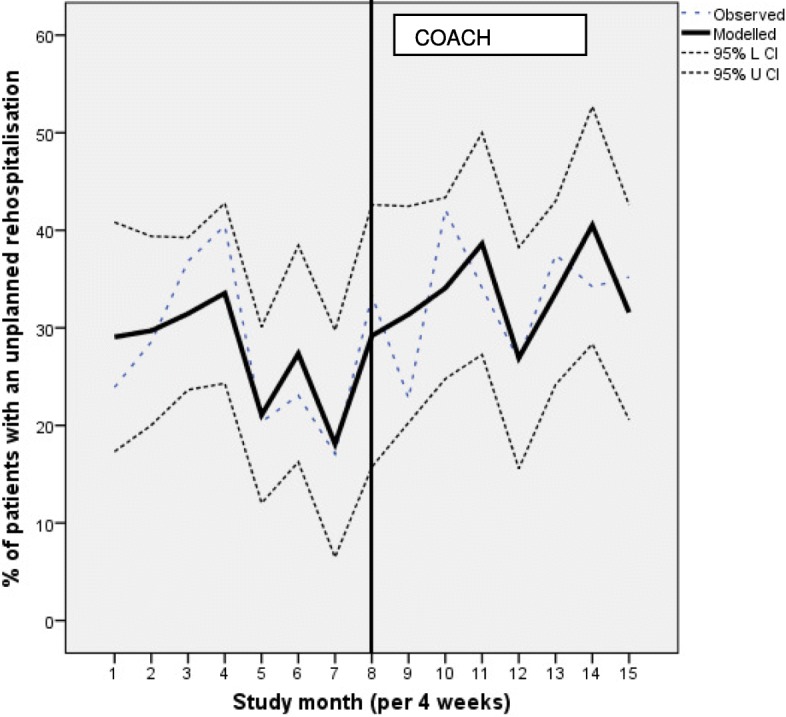
Impact of the COACH program on unplanned rehospitalisations per study month (adjusted for confounders)

In the second segmented linear regression model we adjusted for confounders (help with medication use, all hospital contacts in the last 6 months, mean Charlson score), but again non-significant results were found. β_1_ became − 2.1% (95% CI -5.2; 1.1), β_2_ increased to 12.7% (95% CI -7.3; 32.7) and β_3_ was − 0.2% rehospitalisations per 4 week period (95% CI -4.9-4.6), see Fig. 3 for the proportion of patients with an unplanned rehospitalisation per study month.

### Clinical outcomes

The proportion of patients with any rehospitalisation, planned rehospitalisation and emergency department visits did not differ (Table 4). Also, mortality did not differ (7.6% usual care vs 6.6% COACH program).

**Table 4:**
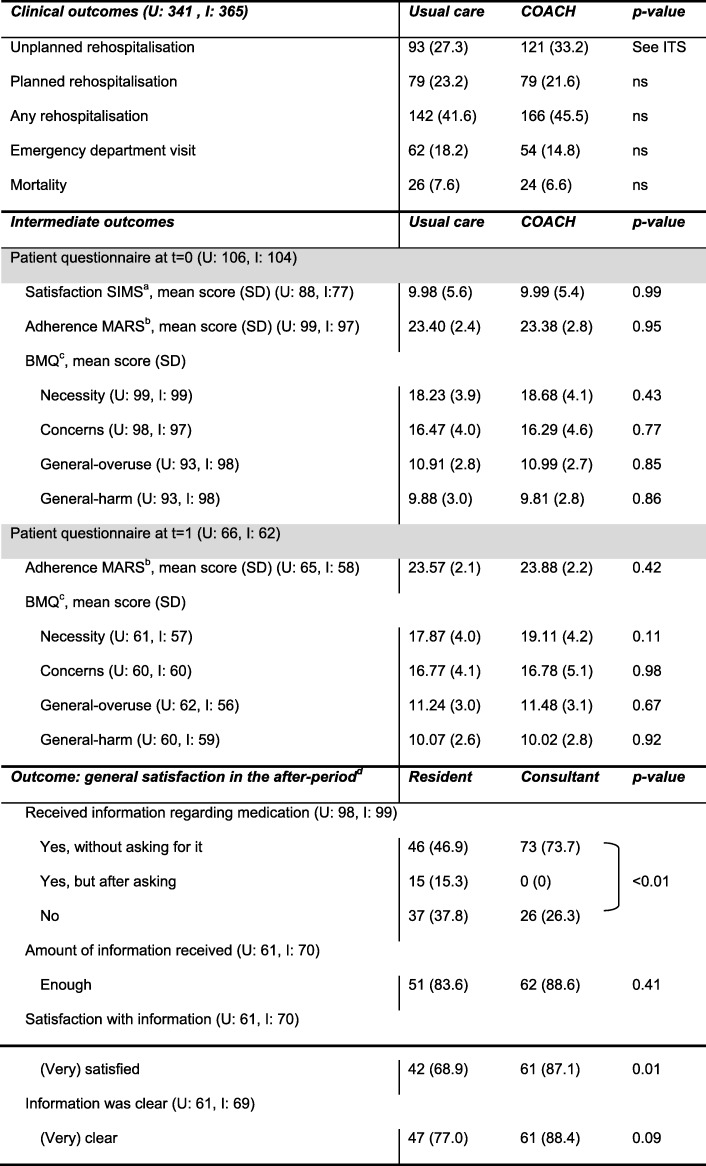
Results of clinical outcomes and intermediate outcomes (patient questionnaires)

U = usual care: number of patients, I = intervention: number of patients, ITS = interrupted time series analysis, t = 0: at discharge, t = 1: 1 month after discharge

^a^Satisfaction with Information about Medicines Scale (SIMS). Higher scores indicate a higher degree of overall satisfaction (17 items: score range 0–17) [29].

^b^Self-report Medication Adherence Rating Scale (MARS). Higher scores indicate higher adherence (5-items: score range 5–25) [30, 31].

^c^Beliefs about medication (BMQ). BMQ-necessity: higher scores indicate beliefs about the necessity and efficacy of medicines (5 items, score range 5–25). BMQ concerns: higher scores indicate concerns about the harmful effects of medicines (6 items, score range 6–30). BMQ General-overuse and BMQ General-harm: higher score indicate beliefs that medicines are over-used by doctors and are harmful addictive poisons (both 4 items, score range 4–20) [27, 28].

^d^Patient’s general satisfaction with counselling by the resident did not significantly differ between the before- and after-period

### Intermediate outcomes

In 100% of patients at least one intervention was recorded aimed at preventing DRPs (mean: 10/patient, Table 5). Medication reconciliation resulted in an average of 5.9 medication changes per patient: 3.9 due to discrepancies between prescribed and actual medication use and 2 due to optimizations in the pharmacotherapy (e.g. discontinuation of hypnotics that were initiated in the hospital). During patient counselling a mean of 4 interventions were aimed to optimise the patient’s medication handling (e.g. answer questions regarding side effects, discuss adherence).

**Table 5:** Effect of the COACH program on medication reconciliation interventions (*n* = 365)

Outcome: drug-related problems	Hospital admission mean/pat (%^d^)	Hospital discharge mean/pat (%^d^)	Patient counselling mean/pat (%^d^)	Total mean/pat (%^d^)
Elimination of discrepancies^a^	1.65 (62.4)	1.43 (68.2)	0.82 (49.7)	*3.90 (89.2)*
Optimisation of pharmacotherapy^b^	0.10 (9.7)	1.76 (75.1)	0.15 (13.0)	*2.02 (80.4)*
Optimisation medication handling^c^	–	–	4.15 (97.8)	*4.15 (97.8)*
Total	*1.75 (64.1)*	*3.19 (93.4)*	*5.12 (98.9)*	*10.07 (100.0)*

^a^ Examples: omission of pre-admission used diabetes drug started at hospital admission, temporarily discontinued anticoagulant restarted at hospital discharge, patient used a different dose of inhalation medication pre-admission

^b^ Examples: a laxative added to opioid use at admission, analgesics or protonpumpinhibitor discontinued at discharge as there was no indication anymore, patient states that sedative is no longer needed

^c^ Examples: questions of patient regarding side effect answered, adherence to medication and helping tools discussed, medication changes explained

^d^ Percent of patients for whom at least one intervention was registered

The response rate for the questionnaires were low (despite telephone calls). There was no significant difference found between groups for adherence, beliefs about medication, and the satisfaction with information about medication (see Table 4). Patients were significantly more satisfied with the information provided by the pharmaceutical consultant (68.9% vs 87.1%, *p* = 0.01).

### Post-hoc analysis: drug-related revisits

Twenty-nine usual care patients (8.5%) had a total of 34 drug-related revisits versus 37 COACH program patients (10.1%) with a total of 44 visits. The reviewers regarded 10 of 34 (29.4%) visits of usual care patients preventable by the COACH program; 4 visits were due to medication reconciliation problems and 6 visits due to an adherence problem. For the COACH program patients 7 of 44 (15.9%) visits were regarded potentially preventable: 1 visit due to a medication reconciliation problem and 6 due to a possible adherence problem. The remaining visits were regarded non-preventable (e.g. side effects, worsening conditions, medication changes implemented post-discharge).

## Discussion

This study showed that the COACH program did not decrease unplanned rehospitalisations. The program identified interventions to prevent DRPs for every included patient. Also, patients expressed a greater satisfaction with the counselling performed. No effect was seen on other exploratory outcomes. Drug-related visits did not differ also, although the number of visits that were potentially preventable with the COACH program decreased from 29.4% in the before-period to 15.9% in the after-period.

Although various transitional pharmaceutical care studies showed a reduction in number of rehospitalisations, our study adds to the studies that did not. This leaves thus an overall mixed picture of the effect of these solely pharmaceutical programs [11, 37, 39–47]. Evidence on components effective for specific pharmaceutical transitional care programs is limited [48, 49].

There may be several reasons for our findings. First, we did not define a high-risk group but included all patients. Scullin et al. reported an 8% reduction in the rehospitalisation frequency after one year in a RCT with a pre-defined high-risk group (49% control vs 41% intervention, *p* = 0.027) [37]. We chose to include all patients because from a patient safety perspective every patient should receive medication reconciliation to prevent medication errors and patient counselling to prevent misunderstanding of the medication changes in the hospital. However, with the current knowledge, a larger sample size was needed.

Second, the implementation fidelity for the COACH program was low for informing the general practitioner through the discharge letter. A study showed decreased 30-day readmission rates (odds ratio 0.61, 95% CI: 0.42–0.88) for patients in whom the intervention was implemented completely in the USA [50]. No decrease was seen for patients who received only parts of the intervention. In the COACH program, the sample size was too small to perform relevant subgroup analysis. Fidelity with informing the general practitioner with the exact same information as was communicated to the patient and community pharmacist was poorly performed by the residents (27.9% of patients). Every resident received the study protocol, training in how to insert the discharge medication into the discharge letter, and feedback during the study. However, the turn-over of residents was high, the residents worked on several departments, and had many tasks or were unaware that general practitioners want to be completely informed [7, 51]. The residents used the standard link to include discharge medication into the discharge letter that was a copy of the medication list at discharge (without allergies and reason for medication changes). However, these allergies were already present before hospital admission so we do not think that allergies were missed by the general practitioner. Furthermore, the fidelity with informing the community pharmacist was 100%. Changes made by the community pharmacist in the patient’s medication record is automatically communicated electronically to the patient’s general practitioner. The general practitioner could miss the reasons for medication changes if the community pharmacy did not document this in their information system.

Third, our intervention might have been too narrow. Previous studies with benefits on rehospitalisations had a broader intervention (e.g. including also post-discharge interventions), did not focus solely on the pharmacotherapy (e.g. interventions on appointment schedules), or used a combination of healthcare providers [35, 37, 50, 52–54].

Fourth, the rehospitalisation outcome was unrealistic. It makes more sense that a program such as COACH only influences drug-related visits, drug-related problems, adverse drug events or general healthcare usage by patients, e.g. additional visits to the community pharmacy or general practitioner for questions or problems with medication. Two studies with transitional pharmaceutical care interventions reported no reduction in overall rehospitalisations but a significant reduction in drug-related rehospitalisations [42, 46]. We saw a non-significant decrease in hospital visits due to a medication reconciliation problem, but this was only shown in a post-hoc analysis and our study was underpowered for this outcome. We chose rehospitalisations as the primary outcome, because policy makers are more interested in clinical outcomes than in intermediate outcomes [55].

Finally, it is unknown to what degree the quality of care after hospital discharge influences outcomes. For example, we have shown that community pharmacies and general practitioners fail to update their patient records with discharge medication related information [56, 57]. This can result in renewed prescribing of previously discontinued medication [6, 58, 59].

Previous studies also showed mixed results for adherence [11, 39, 60–63]. In this study, patients reported very high medication adherence with the MARS questionnaire which could lead to a ceiling effect. But it is more likely that the one-time patient counselling at discharge was not enough to improve the intermediate outcomes such as adherence and beliefs about medication. Patients were more satisfied with counselling by the pharmaceutical consultant than the counselling by the resident. This result corresponds with a previous qualitative study that we performed [64].

The strength of this study was that we assessed rehospitalisations to 6 hospitals, performed an interrupted-time series analysis and we assessed various outcomes. Limitations of this study also need to be discussed. First, patients in the before- and after-period differed in baseline characteristics. We adjusted for these. However, there may be other confounding factors that we did not measure and therefore could not adjust for (e.g. health literacy). Second, patients who did not give informed consent were significantly older and tended to stay longer in hospital, suggesting that patients who were more severely ill refused to participate. It is expected that these patient are rehospitalised more often, so the rehospitalisation frequency may be underestimated. Third, as this study concerns a monocenter study at one department the generalizability is limited. Fourth, more datapoints for the interrupted time series analysis would be desirable, but studies as ours are labour intensive and the number of observations (patients with/without hospitalisations) per data point had to be manually collected. This is very different to e.g., studies that use electronic health records or claims data. This number of data points and follow up time (8 + 9 =) 17 months was the maximum number feasible within the constraints of our study budget. A recent publication of Jandoc et al. acknowledges there is ‘no gold standard’ but suggests a number of nine data points as a minimum considering variation and expected effect size [65]. The number of nine is now also proposed in the updated EPOC guidance [66]. Our study just falls short of this recommendation. Fifth, we had no control line to check how rehospitalisations changed over time in our region. Hospitals are under pressure to become more efficient and readmissions are regarded a quality indicator. That could decrease the readmission rate over years. However, in the Netherlands, the elderly patient population is increasing which also increases rehospitalisations in this patient group. Sixth, we performed a post-hoc analysis for the drug-related visits. Finally, patients did not want to fill out questionnaires as they considered this as a burden or they were not interested in research. The sample size with respect to the questionnaires was limited and the results may be biased as patients who were more interested in the study might have participated.

Future studies need to assess what effective components are and should assess clinical outcomes that are more sensitive to pharmaceutical care interventions, e.g. drug-related readmissions and adverse drug events. Also, studies need to improve continuity of care after discharge by primary healthcare providers. Future research should first consider the sensitivity of the measured outcome. Decision makers could come to the conclusion that interventions do not work while studies are underpowered and cannot show an effect.

## Conclusions

The transitional care program, COACH, did not decrease unplanned rehospitalisations of Internal Medicine patients. Interventions to prevent DRPs were recorded in all patients and patient satisfaction increased.

The lack of effect on unplanned rehospitalisations could be due to the included population (all patients vs high risk), intervention (pharmaceutical or more comprehensive), follow-up (no post-discharge follow up vs home visits or phone calls implemented post-discharge) and outcome (all rehospitalisations vs drug-related re-hospitalisations).

## Data Availability

The datasets used and/or analysed during the current study are available from the corresponding author on reasonable request.

## Abbreviations

BMQ: Beliefs about Medicines Questionnaire
COACH: Continuity Of Appropriate pharmacotherapy, patient Counselling and information transfer in Healthcare
CP: Community pharmacy
CPOE: Computerized Physician Order Entry
DRPs: Drug-related problems,
ED: Emergency department
GP: General practitioner
ITS: Interrupted time series analysis
MARS: Medication Adherence Rating Scale
MR: Medication reconciliation
PC: Patient counselling
SIMS: Satisfaction with Information about Medicines Scale
